# Congenital Distal Tibiofibular Synostosis: A Case Report of Surgical Management in a Skeletally Immature Patient

**DOI:** 10.1155/cro/8243552

**Published:** 2026-01-18

**Authors:** Suguru Kawanishi, Yohei Tomaru, Takashi Saisu, Hiroaki Tsuruoka, Nobuto Kitamura, Makoto Kamegaya

**Affiliations:** ^1^ Department of Orthopaedic Surgery, Chiba Child & Adult Orthopaedic Clinic, Chiba, Japan; ^2^ Department of Orthopaedic Surgery, St. Luke’s International Hospital, Tokyo, Japan, luke.ac.jp

## Abstract

Congenital distal tibiofibular synostosis is quite a rare disease, and little is known about its optimal management. A 6‐year‐old girl presented to our institution with a deformity of her left lower leg. After years of careful observation, the patient underwent partial resection of a laterally protruding portion of the distal diaphysis of the fibula at age 10 for cosmetic reasons. Since the recurrence of lateral prominence of the distal lower leg was observed following the initial surgery, complete resection of the protruding portion of the distal diaphysis of the fibula was performed at age 14. Although mild varus deformity of the ankle was observed, the ankle joint congruity was good, and there were no associated symptoms or functional limitations. Therefore, we decided to perform a simple resection of the distal fibular diaphysis only for cosmetic improvement. At 1‐year follow‐up, no recurrence of lateral prominence of the distal lower leg has been observed. There was no difference in ankle range of motion between the left and right sides. The patient reported no pain and was able to participate in sports activities. In patients with preserved ankle function and few complaints about their daily activities, waiting until skeletal maturity and removing only the affected area may provide sufficient outcomes without risking growth potential or ankle function in the treatment of congenital distal tibiofibular synostosis.

## 1. Introduction

Tibiofibular synostosis, a rare condition in which bony bridges form between the tibia and fibula can cause growth disturbance and malalignment. Distal tibiofibular synostosis has often been reported to be acquired either traumatically or iatrogenically, and a congenital presentation is even rarer [1, 2]. Thus, literature reporting congenital distal tibiofibular synostosis has been scarce to this date, and little is known about its optimal management [3–7]. We present a case of congenital distal tibiofibular synostosis, which was managed surgically.

## 2. Case Presentation

**Figure 1:**
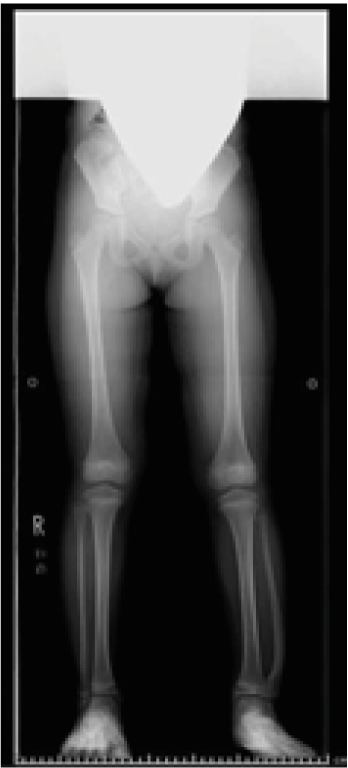
The whole leg radiograph of the patient at the first visit. A bony bridge was noted between the distal tibia and fibula. The distal fibula was laterally protruding.

**Figure 2:**
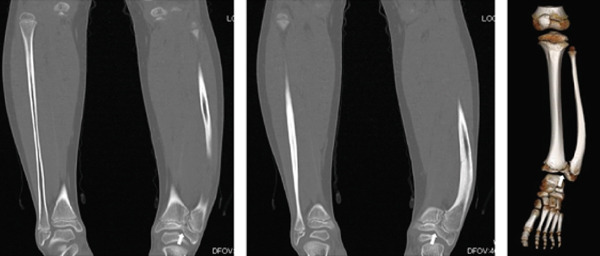
Computed tomography (CT) of lower legs. Epiphyses of the distal tibia and fibula were united (arrow).

A six‐year‐old girl was brought to our institution due to a deformity of the left lower leg noted by her mother. There was no pain or other symptoms besides the deformity, and she had no prior history of trauma, infection, or surgery. No other deformities were noted in the foot or other parts of the body. Functionally, the patient had few problems and was able to perform ballet. Radiographs revealed a bony bridge between the distal tibia and fibula, with the distal fibula protruding laterally (Figure 1). Computed tomography demonstrated synostosis between the distal tibial and fibular epiphyses (Figure 2). Although the physes of the distal tibia and fibula remained open, the distal fibular physis was medially tilting. After a 4‐year follow‐up, the patient and her family requested surgery for cosmetic concerns related to the lateral protrusion of the distal lower leg.

**Figure 3:**
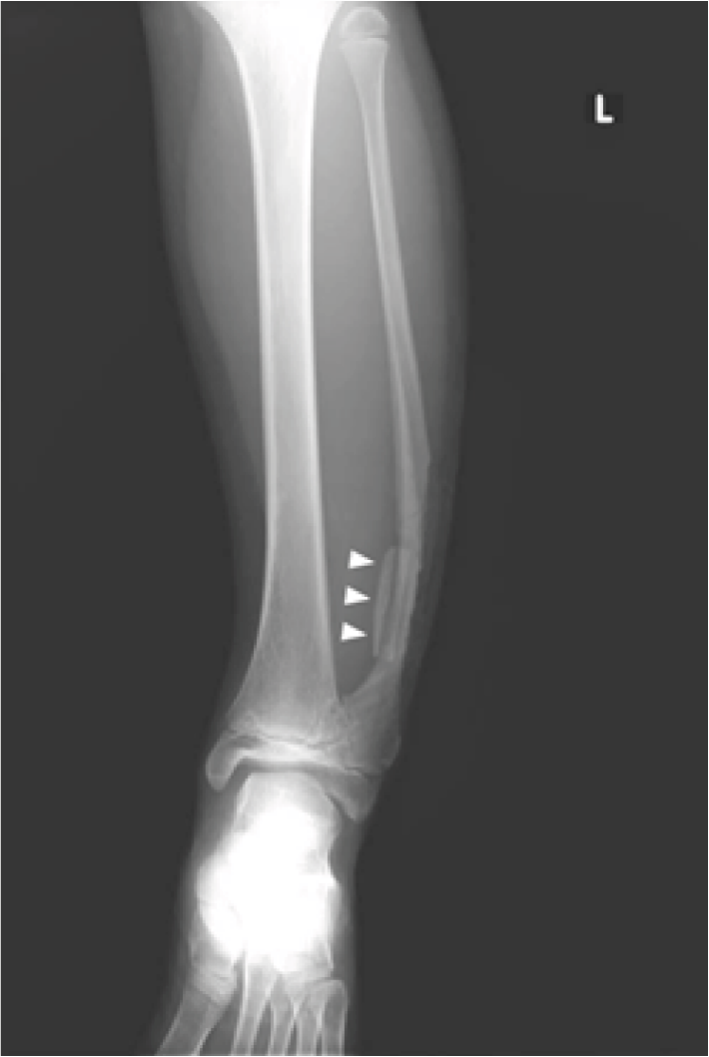
Radiograph after the first surgery, where resection and transfer of the laterally protruding portion of the distal fibula to the medial side were performed (arrowhead).

Preoperative assessment showed varus deformity of the left ankle with a lateral distal tibial angle (LDTA) of 99°; in comparison, the right ankle had an LDTA of 92°. However, the ankle joint congruity was good despite the varus ankle, leading to the decision to proceed with partial resection of the left distal fibula at age 10. A skin incision was made directly upon the lateral prominence of the fibula. A lateral portion of the protruded fibula was dissected and transferred medially, expecting bone remodeling. Bone wax was applied to the osteotomy site (Figure 3).

**Figure 4:**
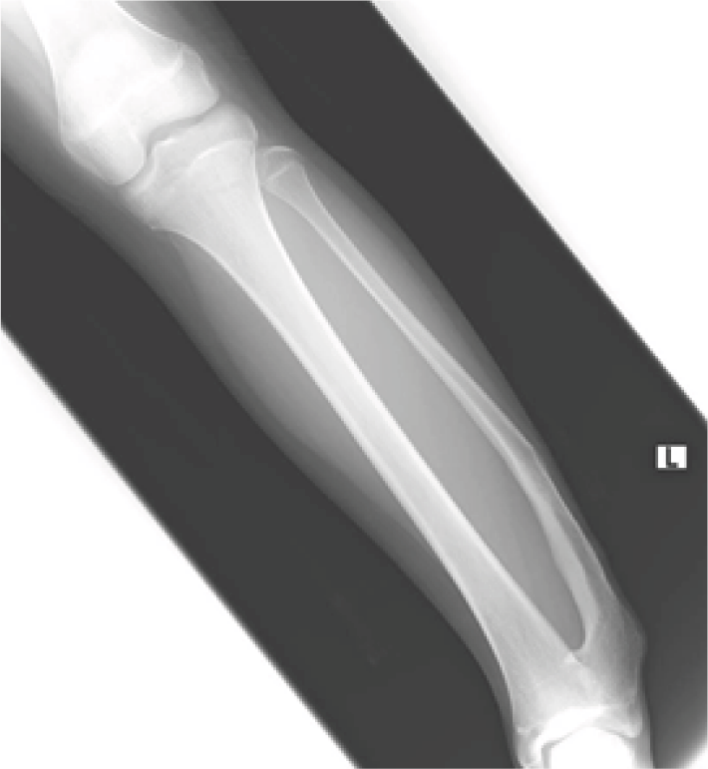
Radiograph 1 year after the initial surgery. The transferred bone was remodeled well, but the lateral protrusion of the fibula recurred.

**Figure 5:**
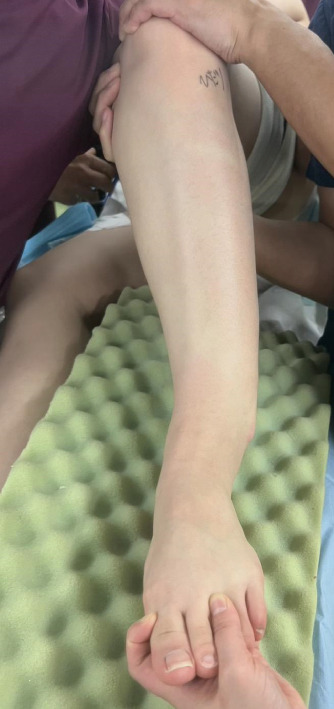
Clinical photo of the patient′s leg before the second surgery. Lateral protrusion of the lower leg was apparent.

**Figure 6:**
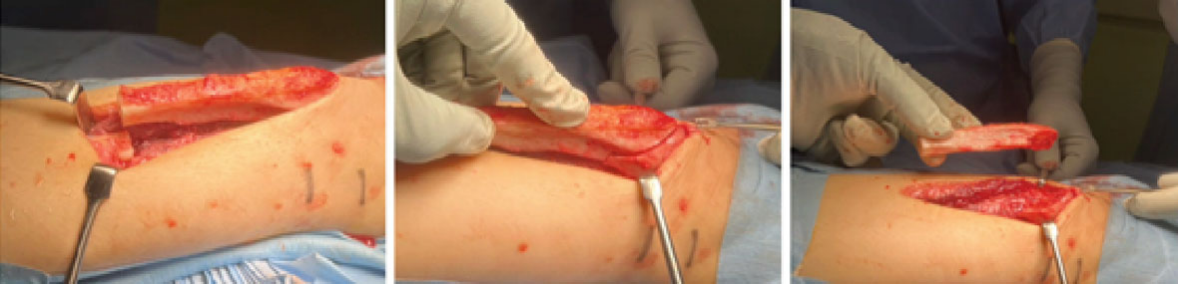
Complete resection of the protruding portion of the fibula was performed. The periosteum was detached from the fibula and preserved, except for the portion resected together with the fibula, which was located superficial to the fibula.

**Figure 7:**
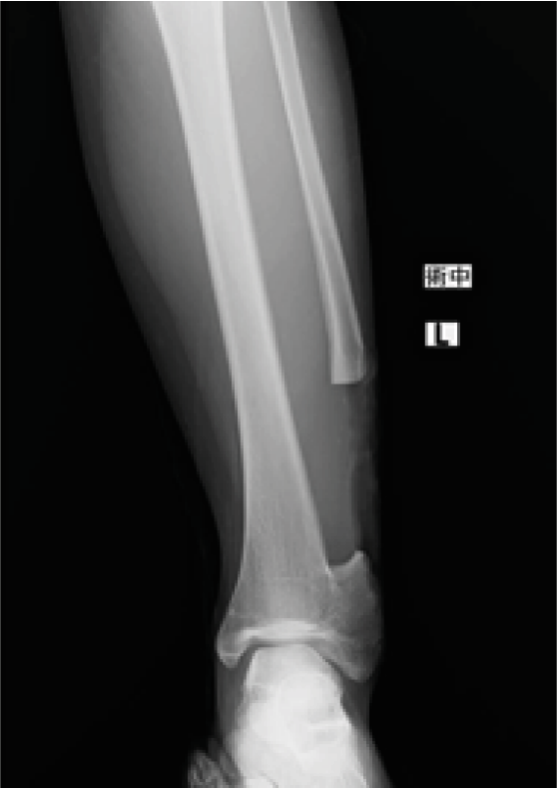
Radiograph after the second surgery. A portion of the distal fibula from the beginning to the end of the lateral bowing was completely resected.

Approximately 1 year postoperatively, although bone remodeling of the graft was achieved, a recurrent lateral protrusion of the fibula was observed (Figures 4, 5). As the lateral protrusion continued to progress, the patient agreed to undergo complete resection of the protruding portion of the fibula at the age of 14. Regarding the periosteum, the portion that is located superficial (lateral) to the fibula was resected along with the fibula to prevent recurrent union and protrusion, whereas the portion that is located deep (medial) to the fibula was preserved for safety, resulting in sub‐periosteal resection of the fibula there (Figures 6, 7).

**Figure 8:**
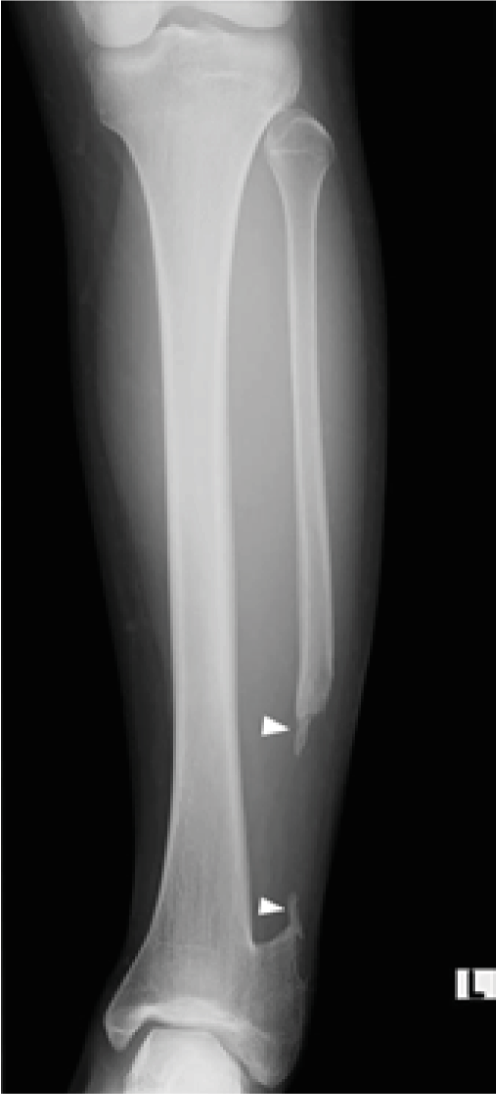
Radiograph 1 year after the second surgery. A little bone extension can be seen from the edges of the resected fibula (arrowhead). There were no signs of lateral bowing, and the distal growth plates of the tibia and fibula had almost closed.

At 1 year following the second operation, the patient reported no pain or difficulty with sports activities, with an ankle range of motion equivalent to that of the contralateral side. On radiographs, bone extension from the edges of the resected fibula can be noted, but cosmetic improvement of lateral prominence was maintained. The distal growth plates of the tibia and fibula had almost closed in the radiogram (Figure 8). Postoperatively, a transient hypoesthesia of the superficial peroneal nerve was observed but improved within 6 months.

Informed consent was obtained from the patient and the patient′s guardian regarding the publication of this case report.

## 3. Discussion

The common pathophysiology of tibiofibular synostosis depends on the location of the fusion, as proximal synostosis is usually congenital, and distal synostosis is often acquired due to trauma or surgery [1, 2]. Congenital distal tibiofibular synostosis, which was considered the diagnosis in the present case, given the absence of trauma or surgery, is an even rarer condition. Although it was reported as early as 1965 by Grobelski M et al., there have been only a few reports to date, especially in the English literature [3–7].

In cases of acquired distal tibiofibular synostosis, a bony bridge is thought to be the result of disruption of the intraosseous membrane and the formation of the hematoma [8, 9]. In healthy patients, because the growth rate of the proximal physis of the fibula is greater than that of the proximal tibia, the fibula elongates (thrusts) down the tibia as it grows, and eventually, the distal end of the fibula will be more distal than that of the tibia [10–12]. Therefore, once the distal tibiofibular joint is fused, the physiologic thrust of the distal fibula does not occur, resulting in a shorter distal fibula and ankle valgus. Moreover, the fibular thrust is reversed and elongated proximally, resulting in protrusion of the fibular head [13].

In the present case, synostosis occurred at the epiphysis, and no bony bridges were observed in the interosseous membrane, which may have prevented the reversal of the thrust and the consequent protrusion of the fibular head. On the other hand, the distal tip of the fibula was located more proximally than the contralateral side, possibly because the fibula cannot extend distally beyond the synostosis. Instead, the distal fibula had extended laterally, potentially due to the tilted growth plate, resulting in the lateral protrusion. Jung et al. noted similar findings in their case report of congenital distal tibiofibular synostosis [5].

For acquired distal tibiofibular synostosis, several treatment options, ranging from conservative measures to surgical excision of the synostosis, have been reported. Generally, patients with acquired distal tibiofibular synostosis have few complaints and do not require any interventions, and surgery serves as a last resort when the pain or limitation of motion cannot be tolerated [14–16]. In skeletally immature children, follow‐up until skeletal maturity is reasonable, with attention to growth disturbances and malalignment [9, 12].

In contrast, there has been scarce literature describing congenital distal tibiofibular synostosis itself, let alone its optimal management and prognosis. To the best of our knowledge, there has been only one report regarding surgically treated congenital distal tibiofibular synostosis, written in Korean. Jung et al. described their surgical procedure as transferring a resected portion of the distal fibula to the anatomical position, aiming for ankle mortise reconstruction. They reported that good activity and range of motion were achieved, although additional surgery was required due to nonunion [5]. This proposed procedure, however, appears to be technically demanding and carries a risk of harming the growing potential or anatomical alignment, as it requires resection of the opening physis and repositioning it distally.

In our case, although surgery was eventually performed for cosmetic reasons, the lateral malleolus of the ankle was only slightly shorter than the medial one, and both the ankle mortise and function were preserved, which warranted several years of observation. Initially, we decided to perform a partial resection of the distal fibular diaphysis for the following reasons: first, complete resection of the distal fibula would disrupt the ankle mortise, which might compromise joint stability and function; second, by resecting the connection of the proximal and distal fibula, which might previously have anchored to each other, bone growth from the stumps could deviate laterally, resulting in subsequent lateral prominence; and third, complete resection would remove the fibular attachment of the flexor hallucis longus, potentially impairing the patient′s ballet performance. Following the surgery, however, although remodeling of the transferred bone was achieved, lateral bowing of the distal fibula recurred because growth potential remained, necessitating the second surgery.

Upon the second surgery, decisions had to be made regarding the surgical plan among partial resection, complete resection, and ankle reconstruction. Among these options, complete resection was chosen in order to achieve cosmetic improvement while minimizing the risk of recurrence. On the other hand, since there were no symptoms, including pain, instability, or limited range of motion, aggressive reconstruction of the ankle joint seemed unnecessary. Following the second surgery, although it did not reconstruct the anatomical function of the ankle since the distal fibula was not relocated, it resulted in cosmetic improvement, preserved function, and no recurrence of the lateral bowing at the 1‐year follow‐up.

Since this is a single case report with a short postoperative follow‐up of only 1 year, surgeons should note that treatment strategies for similar cases require thorough discussion, taking into account the patient′s complaints, the degree of deformity, and the risk of growth impairment. However, we believe that resection of the protruding bone, as in this case, is less invasive compared with the previously described procedure by Jung et al., as it does not require handling of the growth plates. In patients with preserved ankle function and few complaints about their daily activities, waiting until skeletal maturity and removing only the affected area may provide sufficient outcomes without risking growth potential or ankle function.

## Conflicts of Interest

The authors declare no conflicts of interest.

## Funding

No funding was received for this manuscript.

## Data Availability

Data sharing not applicable to this article as no datasets were generated or analysed during the current study.
